# The sex-specific effects of diet quality versus quantity on morphology in *Drosophila melanogaster*

**DOI:** 10.1098/rsos.170375

**Published:** 2017-09-06

**Authors:** Alexander W. Shingleton, Josephine R. Masandika, Lily S. Thorsen, Yuqing Zhu, Christen K. Mirth

**Affiliations:** 1 Department of Biology, Lake Forest College, Lake Forest, IL 60045, USA; 2 Division of Biology and Biomedical Sciences, Washington University, St Louis, MO 63110, USA; 3 School of Biological Sciences, Monash University, Clayton, Victoria, Australia; 4 Instituto Gulbenkian de Ciência, Rua da Quinta Grande 6, 2780-156 Oeiras, Portugal

**Keywords:** allometry, dietary restriction, dietary imbalance, fruit fly, nutritional geometry, macronutrient composition

## Abstract

Variation in the quality and quantity of nutrition is a major contributor to phenotypic variation in animal populations. Although we know much of how dietary restriction impacts phenotype, and of the molecular-genetic and physiological mechanisms that underlie this response, we know much less of the effects of dietary imbalance. Specifically, although dietary imbalance and restriction both reduce overall body size, it is unclear whether both have the same effect on the size of individual traits. Here, we use the fruit fly *Drosophila melanogaster* to explore the effect of dietary food versus protein-to-carbohydrate ratio on body proportion and trait size. Our results indicate that body proportion and trait size respond differently to changes in diet quantity (food concentration) versus diet quality (protein-to-carbohydrate ratio), and that these effects are sex specific. While these differences suggest that *Drosophila* use at least partially distinct developmental mechanisms to respond to diet quality versus quantity, further analysis indicates that the responses can be largely explained by the independent and contrasting effects of protein and carbohydrate concentration on trait size. Our data highlight the importance of considering macronutrient composition when elucidating the effect of nutrition on trait size, at the levels of both morphology and developmental physiology.

## 1. Introduction

The developmental regulation of body and trait size is a fundamental process that ensures that traits grow to an appropriate size for the body as a whole, and the body grows to an appropriate size for the environment in which the animal inhabits. Body and trait size are regulated by both genetic and environmental factors, and extensive research over the last 20 years has elucidated how these factors influence the physiological and cellular mechanisms that control growth and development [1]. Of these factors, we know most about the effects of developmental nutrition on adult trait and body size, perhaps because this is one of the most biologically apparent forms of phenotypic plasticity and has been studied for well over 100 years [2,3]. Up to this point, research on body and trait size has largely focused on the quantity, rather than the quality, of nutrition; that is, how the total amount of food influences body and trait size, rather than the composition of the food [1,4–8]. Nevertheless, there is increasing research to demonstrate that dietary balance, specifically the relative amounts of lipids, carbohydrates and proteins in a diet, influences animal growth and adult body size [9–15]. Furthermore, there is a rich literature exploring the effects of C : N : P stoichiometry on growth rate and nutrient cycling in aquatic and terrestrial food webs [16–19], and on mapping various life-history characteristics—including body size—across a nutritional landscape of diets that vary in the quantity and ratio of different macronutrients [20–23]. While these studies reinforce the importance of dietary balance as a regulator of body size, what is unclear is whether the effects on body and trait size generated by changes in diet quality are developmentally equivalent to the effects generated by changes in diet quantity.

This is an important question. Decades of research have been dedicated to elucidating the molecular, genetic and physiological mechanisms through which diet quantity affects growth [1]. These mechanisms are largely conserved among all animals and have been used to explain phenomena such as sexual-size dimorphism, exaggerated secondary sexual characteristics, canalization and the evolution of body size [24–27]. Whether these mechanisms can also be used to explain the effects of diet quality on growth is, however, largely unknown.

In general, dietary restriction during development causes a reduction in adult body and trait size. Not all traits are equally sensitive to changes in dietary quantity, however. Some traits, for example, the horns of male rhinoceros beetle [25] or the antlers of red deer stags [28], are extremely sensitive to changes in the amount of nutrition available during development. Thus, as body size increases with increased nutrition, these traits become proportionally larger so that they are exaggerated in the largest individuals. By contrast, other traits, for example, the central nervous system [29] and male genitalia [26] in *Drosophila melanogaster*, are relatively insensitive to dietary restriction, and are more-or-less the same size in both large, well-fed and small, poorly fed individuals. Because traits differ in their sensitivity to the genetic and environmental factors that regulate size, body proportion changes with overall size, a phenomenon called ‘allometry’. Allometry is typically modelled using the allometric equation: log *y *= log*b *+ *a*log*x*, where *x* and *y* are the sizes of different traits, and *α* is the allometric coefficient [30]. When size varies entirely in response to an environmental factor, the allometric coefficient captures the sensitivity of trait *y* to changes in that factor relative to trait *x*; that is, the relative plasticity of trait *y* [4].

Importantly, however, a trait's sensitivity to changes in one environmental factor is not necessarily a general reflection of its sensitivity to changes in all size-regulatory factors. For example, wing size in *D. melanogaster* is, relative to other traits, much more sensitive to changes in temperature than to changes in developmental nutrition [31]. A consequence of this is that body proportion changes differently in response to variation in rearing temperature compared to variation in developmental nutrition. This, in turn, suggests that temperature and nutrition influence trait size through at least partially distinct developmental mechanisms.

An added nuance is that there are often sex differences in size plasticity. Across insect species, body mass is typically more plastic in females than males in response to a variety of environmental factors, including diet quality, diet quantity and temperature [32,33]. The opposite may be true in vertebrates, where males are often the larger sex [34]. While these sex differences in plasticity are well established, the mechanisms that underlie them are poorly understood. It is known, however, that males and females can differ in their nutritional preferences [35] and in how changes in diet and temperature affect key developmental parameters that regulate body size [36,37]. Thus, there may be differences in how males and females respond to changes in diet quality versus quantity. Interestingly, in insects, the bias for greater female plasticity is removed when other morphometric proxies of body size are used, for example, body length or wing size, with as many species showing higher plasticity in males as in females [32]. This suggests that sex-specific plasticity may vary among traits, where some traits are more plastic in males, while others are more plastic in females. If this were true, we would also expect allometric scaling to be different in males and females. This has been observed in a number of species, particularly those carrying exaggerated male traits, such as stalk-eyed flies or horned-beetle [38,39]. Nevertheless, we have a poor understanding of sex-specific scaling outside of secondary sexual characteristics (although see [40]).

We explored the question of whether diet quality and quantity affect size through the same developmental mechanisms by elucidating their effects on the size of individual traits in males and females. Specifically, if the same developmental mechanisms regulate the effects of diet quality and quantity, then we would expect that traits that are known to be more (or less) sensitive to changes in diet quality would be correspondingly more (or less) sensitive to changes in diet quantity. Furthermore, we would expect that the effect of diet quality on body proportion would be the same as the effect of diet quantity. Finally, given well-established sex-specific differences in phenotypic plasticity, we might expect the effects of changes in diet quality and quantity on body proportion to be different in males and females. Here, we test these hypotheses, using *D. melanogaster* reared on diets with different protein-to-carbohydrate (P : C) ratios (diet quality) and with different total food concentration (diet quantity).

## 2. Material and methods

### 2.1. Fly stocks and culture medium

We used an outbred population of *Drosophila melanogaster* from Azeitão, Portugal, maintained for more than 50 non-overlapping generations [41]. Prior to the experiment, flies were reared in population cages with over 1000 flies per cage on standard media of 45 g of molasses, 75 g of sucrose, 70 g of cornmeal, 20 g of yeast extract, 10 g of agar, 1100 ml of water and 25 ml of a 10% nipagin solution per litre of fly food [41]. Maintaining flies in this manner ensures that we minimize the loss of genetic diversity in our outbred population.

### 2.2. Diet manipulation

Flies developed from eggs to adults on one of 24 diets prepared as described in Rodrigues *et al.* [23]. Diets varied in their sucrose (Sidul, Santa Iria de Azóia, Portugal) and yeast (Lesaffre SAF-Instant Red) concentration to yield diets with six protein-to-carbohydrate ratios, 1 : 14.6, 1 : 7.2, 1 : 3.5, 1 : 1.7, 1.3 : 1, 1.4 : 1, and were prepared at four yeast/sucrose concentrations: 45, 90, 180 and 360 g l^−1^. Diets were prepared by mixing yeast (Lesaffre SAF-Instant Red), containing 44% protein and 33% carbohydrate, with sucrose (Pró-vida, Algueirão, Portugal), both in a solution with 0.5% agar. The food was autoclaved and to each 500 ml of food we added 5 ml of both propionic acid (Acros Organics, Geel, Belgium) and 10% nipagin (10% *p*-hydroxybenzoic acid methyl ester in 95% ethanol) (Apex BioResearch Products). The food was then pipetted into standard 25 × 95 mm vials (5 ml vial^−1^).

We introduced oviposition plates, 60 mm petri dishes filled with standard laboratory food, into the population cages from the outbred population for 4 h. From these dishes, eggs were randomly transferred in batches of 30 into vials containing 5 ml of the treatment food. Each diet was replicated in six vials and each replicate was established on the same day. Cultures were maintained at 25°C in a climate-controlled room at 60–70% humidity.

### 2.3. Morphological measurements

Measurements were collected as described in Shingleton *et al.* [31]. Briefly, we dissected the wing, maxillary palp, the first femur and genital arch (males only) from the right side of each fly (except in a few cases where the right side was damaged). All body parts were mounted in dimethyl hydantoin formaldehyde. We measured the area of the wing, maxillary palp and posterior lobe of the genital arch, and the length of the femur using a Leica DM6000B compound microscope and Retiga 200R digital camera. We measured the length of the thorax from where the neck meets the pronotum to the posterior tip of the scutellum, using a Leica MZ16FA and Leica DFC250 digital camera. Figure 3 shows the measurements made on each body part. Electronic supplementary material, table S1, gives the number of flies dissected for each diet. On average, we dissected 17 flies per each sex per diet and no more than 30 flies from any one vial. For some of the more extreme diets, this number was substantially lower due to reduced survival. Image processing was conducted using ImagePro v. 6.1. Measurement error is reported in [31].

### 2.4. Statistical analysis

All analyses were conducted using R (http://www.R-project.org/). Linear measurements were squared prior to analysis to convert them to the same dimension as area measurements. All the data were then log transformed. This renders variation in trait size scale-independent, and allows meaningful comparison of parameters from the same statistical model applied to different traits [42]. All the data and R scripts for the analyses are archived in Figshare (https://figshare.com/s/e17afa5d21ffbb1daf69). Where necessary, *p-*values were adjusted for multiple comparisons using a Bonferroni correction.

### 2.5. Univariate analysis

Nonlinear response surfaces are routinely modelled using the second-order polynomial regression [22,23,43–45]. To determine the effect of P : C ratio and food concentration on each trait's size, we therefore fitted the model:

$$S_{ijkl}=F_{i}+K_{j}+F_{i}^{2}+K_{j}^{2}+F_{i}K_{j}+V_{k}+\varepsilon_{ijkl},$$

where *S* is log-transformed trait size, *F* is food concentration (continuous), *K* is P : C ratio (continuous), *V* is replicate vial (random effect) and *ϵ* is error (subscripts are levels within variables). Models were fitted using the *nlme* package in R [46], and marginal *R*^2^ were calculated for the model using the *piecewiseSEM* package in R [47]. For each trait, the significance of each parameter was tested with an ANOVA and any non-significant parameters were subsequently removed from the model before the data were reanalysed using the simplified model. To test whether the response to changes in food concentration and P : C ratio varied between morphological traits we fitted two models:

$$\begin{array}{rl} \text{Model }1\;:\;S_{ijklm} & =T_{l}\times (F_{i}+K_{j}+F_{i}^{2}+K_{j}^{2}+F_{i}K_{j})+V_{k}+\varepsilon_{ijklm}\\ \;\text{and}\;\;\text{Model }2\;:\;S_{ijklm} & =T_{l}+(F_{i}+K_{j}+F_{i}^{2}+K_{j}^{2}+F_{i}K_{j})+V_{k}+\varepsilon_{ijklm}, \end{array}$$

where *T* is trait type*.* The models were compared using a log-likelihood ratio test. If inclusion of trait type as an interactive factor rather than an additive factor significantly improved the fit of the model, we concluded that the relationship between food concentration and P : C ratio varied between traits. We used the same method to test whether the response of trait size to changes in diet was different between males and females.

To determine the individual effect of protein and carbohydrate concentration on each trait's size, we fitted the model:

$$S_{ijkl}=C_{i}+P_{j}+{C_{i}}^{2}+{P_{j}}^{2}+C_{i}P_{j}+V_{k}+\varepsilon_{ijkl},$$

where *C* is carbohydrate concentration (continuous), *P* is protein concentration (continuous) [22]. As before, the model was simplified if any parameter values were found to be non-significant. The final model explaining the effect of protein and carbohydrate levels on trait size was then visualized as a response surface. Although tests of carbohydrate and protein level on trait size are equivalent to tests of P : C ratio and food concentration (because the carbohydrate and protein levels can be calculated from P : C ratio and food concentration, and vice versa), the latter allows the specific effects of P : C ratio and food concentration to be examined, while the former emphasizes the effect of each individual macronutrient.

For all analyses, all independent variables (apart from ‘vial’) were treated as continuous factors. Consequently, while we had low sample sizes for some individual diets (electronic supplementary material, table S1), we had large sample sizes to detect trends across the full range of dietary conditions (413 females and 352 males). For all analyses, we plotted residual against fitted values to confirm homogeneity of variance and generated a QQ plot to confirm that the residuals were approximately normally distributed.

### 2.6. Multivariate allometric analysis

To determine how body proportion changes in response to changes in diet quality versus quantity, we calculated the multivariate allometric coefficients of each trait when body size varied with food concentration at each P : C ratio, and when body size varied with P : C ratio at each food concentration*.* We refer to the former as *food concentration allometries* and the latter as *P : C allometries.* For multivariate data, the multivariate allometric coefficients are the coefficients of the first principal component (PC) of the variance–covariance matrix, also called the ‘allometric vector’ [48,49]. Multiplying the PC coefficients by √*n*, where *n* is the number of traits, gives the bivariate allometric coefficient for each trait against a multivariate measure of overall body size. Because there was a considerable amount of size variation among flies reared in the same vial, the allometric coefficients were calculated from the mean trait sizes for each replicate vial at each P : C ratio and food concentration. We used a random-variable bootstrap method to estimate the precision of each coefficient, based on 10 000 bootstrap samples, as described by Shingleton *et al.* [31].

We used a common slope test to test whether bivariate allometric relationships were significantly different from each other, using the *smatr* package in R [50]. To compare multivariate allometries generated under different conditions, we calculated the angle between pairs of allometric vectors, using the *arc cosine* of their inner product. The larger this angle, the more different the allometric coefficients [31,49]. Because one can only compare allometric vectors with the same number of dimensions, we did not include male genital size in any comparison between male and female allometries. We used a permutation test to establish whether the angle was significantly greater than expected under the null hypothesis that the observed data share the same multivariate allometry, as described in [31]. Because we measured six food concentration plasticities and four P : C plasticities in each sex, this generated 190 pairwise comparisons. We then used Fisher's method [51] to conduct a meta-analysis of all pairwise plasticity comparisons between food concentration and P : C plasticities within sexes, between males and females within each type of plasticity, and among plasticities of the same type within each sex.

## 3. Results

### 3.1. Size response to diet quality and quantity varies among traits and between sexes

For almost all traits in both sexes, both food concentration and P : C ratio significantly affected trait size (table 1). In males and females, there was a positive linear relationship between food concentration and size, but a negative quadratic relationship between P : C ratio and size. Thus, trait size increased with food concentration but decreased with diets that had an excess of either protein or carbohydrates. In all female traits, and in the male femur, wing and genitalia, there was also a significant interaction between food concentration and P : C ratio, such that the effect of one depended on the level of the other. In both sexes, however, traits varied in their response to changes in diet quality and quantity (tables 2 and 3). This was most obvious in the male genitalia, the size of which was largely insensitive to changes in both P : C ratio and food concentration (table 1). The dietary response of the male genitalia was significantly different to the response of the femur, thorax and wing (table 2). All the female traits differed in their response to a change in diet, apart from the wing and the palp (table 3).

**Table 1.** Effects of food concentration (*F*), P : C ratio (*K*) and their squares and products on organ size in males and females. For all traits, the data were fitted with linear mixed-effects models by restricted maximum likelihood. Non-significant parameters were removed from final model, unless their removal rendered the higher order factors non-significant. *p < 0.05, **p < 0.01, ***p < 0.001. Marginal *R^2^* is based on fixed effects only.

body part	sex		*F*	*F*^2^	*K*	*K*^2^	*F* × *K*	marginal *R*^2^
femur	male	*β*	0.0005		0.4740	−0.2155	−0.0003	0.283
		*t*-value	4.048***		5.113***	−3.650***	−2.525*	
	female	*β*	−0.0003	0.000002	0.6077	−0.2685	−0.0005	0.383
		*t*-value	−0.619	2.146*	7.304***	−5.286***	−4.283***	
palp	male	*β*	0.0002		0.2892	−0.1627		0.090
		*t*-value	2.056*		2.599*	−2.205*		
	female	*β*	0.0005		0.6389	−0.2832	−0.0005	0.220
		*t*-value	4.735***		5.396***	−3.827***	−4.057***	
thorax	male	*β*	0.0003		0.3569	−0.1712		0.212
		*t*-value	3.066**		2.857**	−2.325*		
	female	*β*	0.0005		0.6320	−0.2735	−0.0005	0.394
		*t*-value	5.502***		7.955***	−5.670***	−4.285***	
wing	male	*β*	0.0002		0.3400	−0.1541	−0.0002	0.221
		*t*-value	3.827***		3.604***	−2.957**	−2.130*	
	female	*β*	0.0005		0.5039	−0.2208	−0.0002	0.385
		*t*-value	6.318***		8.731***	−6.307***	−5.703***	
genital	male	*β*	0.00002	−0.000001	0.1931	−0.0805	−0.0002	0.031
		*t*-value	2.056*	−1.729	2.238*	−1.515	−2.125*

**Table 2.** Differences in the size response to changes in diet among male traits. Models including trait as an additive versus interactive factor were compared using partial *F*-tests. Models where the interaction significantly improved the fit, and hence where the other parameters of the model differed between traits, are highlighted in italics. The *p*-values were adjusted for multiple comparisons using the Holm method.

trait A	trait B	degrees of freedom	*L* ratio	adjusted *p*-value
wing	palp	5	8.170	0.589
wing	femur	5	4.334	1.000
wing	thorax	5	1.849	1.000
*wing*	*genital*	*5*	*30.376*	*<0.001*
palp	femur	5	14.996	0.073
palp	thorax	5	14.387	0.080
palp	genital	5	9.361	0.477
femur	thorax	5	6.941	0.675
*femur*	*genital*	*5*	*45.439*	*<0.001*
*thorax*	*genital*	*5*	*36.853*	*<0.001*

**Table 3.** Differences in the size response to changes in diet among female traits. Models including trait as an additive versus interactive factor were compared using partial *F*-tests. Models where the interaction significantly improved the fit, and hence where the other parameters of the model differed between traits, are highlighted in italics. The *p*-values were adjusted for multiple comparisons using the Holm method.

trait A	trait B	degrees of freedom	*L* ratio	adjusted *p*-value
wing	palp	5	3.033	0.694
*wing*	*femur*	*5*	*22.177*	*0.002*
*wing*	*thorax*	*5*	*25.593*	*0.001*
*palp*	*femur*	*5*	*16.340*	*0.017*
*palp*	*thorax*	*5*	*24.360*	*0.001*
*femur*	*thorax*	*5*	*16.452*	*0.017*

For all traits, females were more responsive to changes in diet than males, indicated by consistently higher parameter values for the statistical models fitted to female data versus male data (table 1). Furthermore, for all traits, variation in diet explained more variation in trait size in females than in males, evident from the higher *R*^2^ for these statistical models in females versus males (table 1). Direct comparisons of the response of females versus males for each trait indicate a significant difference between the sexes for all traits except for the palp (table 4).

**Table 4.** Differences in the size response to changes in diet between males and females for the same trait. Models including sex as an additive versus interactive factor were compared using partial *F*-tests. Models where the interaction significantly improved the fit, and hence where the other parameters of the model differed between sexes, are highlighted in italics.

trait	degrees of freedom	*L* ratio	*p*-value
*wing*	*5*	*18.065*	*0*.*003*
palp	5	9.600	0.087
*femur*	*5*	*21.948*	*less than 0.001*
*thorax*	*5*	*13.793*	*0*.*017*

### 3.2. Diet quality affects body proportion differently from diet quantity

**Figure 1.**
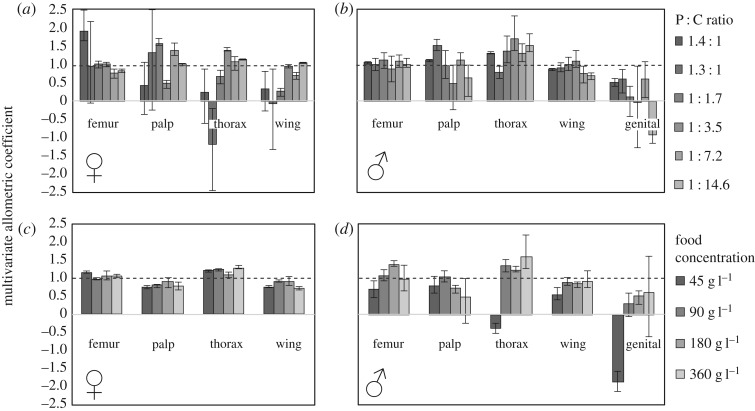
Multivariate allometric coefficients for female and male traits when size varies with food concentration at different P : C ratios (*a*,*b*) and with P : C ratio at different food concentrations (*c*,*d*). The allometric coefficients are standardized such that a coefficient of 1 indicates a trait scales isometrically to body size (horizontal dashed line). Error bars are 95% CI calculated from 10 000 bootstrap samples.

We used multivariate analysis to calculate the allometric coefficients for the relationship between each trait and overall body size, when body size varied in response to changes in food concentration at each P : C ratio (food concentration allometries; figure 1*a*,*b*), and when body size varied in response to changes in P : C ratio at each food concentration (P : C allometries; figure 1*c*,*d*). The allometric coefficient captures the relative sensitivity of each trait to each environmental variable, for example, variation in P : C ratio at a food concentration of 90 g l^−1^. Consistent with our univariate analysis, sensitivity varied among traits for the same environmental variable (figure 1). In general, the male genitalia were the least sensitive to changes in both P : C ratio and food concentration, while the thorax was the most sensitive in both sexes, followed by the femur, the palp and the wing (figure 1). However, the multivariate analysis also indicates that for many traits, their relative sensitivity to changes in food concentration varied with P : C ratio. This echoes the significant interaction between the effect of P : C ratio and food concentration on size for all female traits and the male wing, femur and genitalia (table 1).

**Figure 2.**
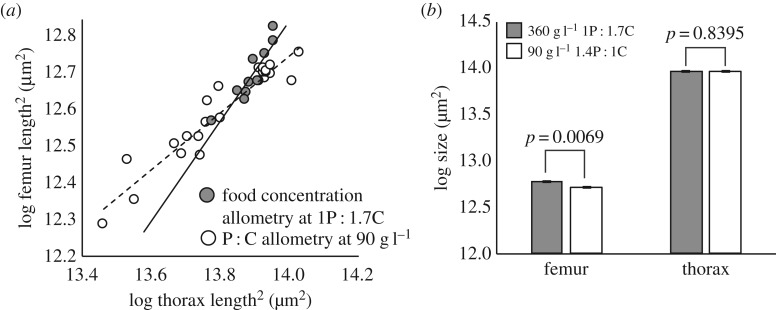
The effect of food concentration and P : C ratio on morphological scaling. (*a*) The relationship between wing and thorax size is different when P : C ratio changes at a fixed food concentration of 90 g l^−1^ (open circles, broken line) than when food concentration changes at a fixed P : C ratio of 1 : 1.7 (closed circles, solid line; common slope test, *p* *<* 0.0001). Each point is a mean wing and thorax size for flies reared in the same vial. (*b*) Flies reared at 360 g l^−1^ and 1 : 1.7 P : C ratio had the same thorax size as flies that were reared at 90 g l^−1^ and a 1.4 : 1 P : C ratio but had significantly longer femurs (linear mixed model, *S_ij_* = *D_i_* *+* *v_ij_*, where *S* = trait size, *D* = diet and *v* = vial). Error bars are 95% CI.

When traits vary in their sensitivity to an environmental regulator of size, there is a change in body proportion as body size changes across an environmental gradient. If diet quality affects trait size in the same way as diet quantity, then body proportion should change in the same way in response to changes in both P : C ratio and food concentration. This was not the case. For example, in females the allometric relationship between femur and thorax size was steeper when food concentration varied (at a P : C ratio of 1 : 1.7) than when P : C ratio varied (at a food concentration of 90 g l^−1^; common slope test, *p* *<* 0.0001; figure 2*a*). Consequently, female flies that were reared at 360 g l^−1^ and a 1 : 1.7 P : C ratio had the same thorax size as flies that were reared at 90 g l^−1^ and a 1.4 : 1 P : C ratio (*p* = 0.8395), but had significantly longer femurs (*p* = 0.0069; linear mixed model, *S_ij_* = *D_i_* *+* *v_ij_*, where *S* = trait size, *D* = diet and *v* = vial; figure 2*b*).

To more formally compare multivariate allometries, we calculated the angle between the allometric vectors for each pair of allometries (190 pairwise comparisons, table 5). This is equivalent to calculating the angle between the two slopes in figure 2*b*, except in four and five dimensions (femur, palp, thorax, wing and genitals). If diet quality and quantity affect trait size in the same way, then the angle between the P : C allometries and the food concentration allometries should not be significantly different from zero. By contrast, when the angle is very large, this indicates that the response of body proportion to changes in P : C ratio is different from the response to changes in food concentration. A meta-analysis of the pairwise comparisons revealed that, in both sexes, P : C allometries were different from food concentration allometries (Fisher's method, *p* < 0.0001 for both, table 5, blue cells; electronic supplementary material, table S2).

**Table 5.** Pairwise comparisons of allometries generated in response to variation in caloric value (food-concentration allometries) at different P : C ratios and in response to variation in P : C ratio (P : C allometries) at different food concentrations, in males (♂) and females (♀). Angles between allometric vectors are shown above the diagonal and uncorrected *p*-values are shown below the diagonal. Values of *p* of less than 0.05 are shown in bold. The darker the cell the lower the *p*-value. Green cells show comparison among food-concentration allometries within each sex; purple cells show comparison among P : C allometries within each sex; blue cells show comparison between food-concentration and P : C allometries within each sex; and red cells shows comparisons between male and female allometries.

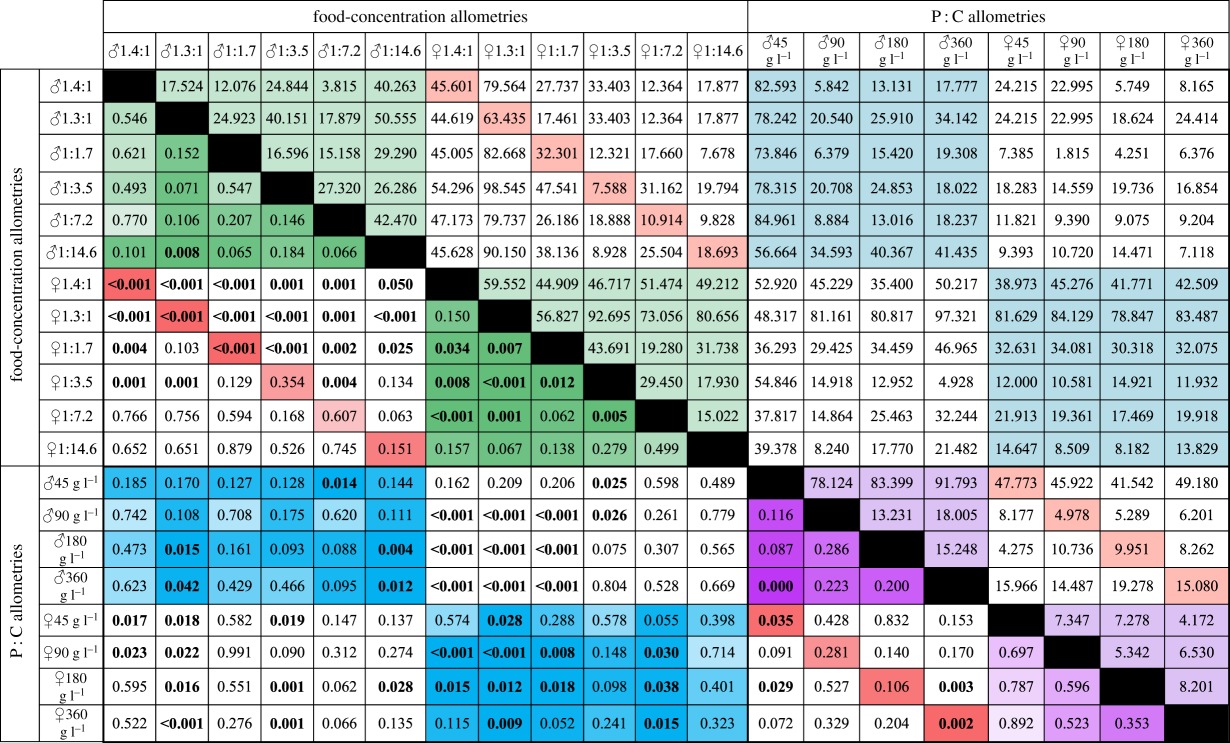

We also found that P : C allometries varied with food concentration in males (Fisher's method, *p* *=* 0.0016, table 5, purple cells; electronic supplementary material, table S3), but not in females (Fisher's method, *p* *=* 0.9234, table 5, purple cells; electronic supplementary material, table S3), while food concentration allometries varied with P : C ratio in both sexes (Fisher's method, *p* < 0.005 for both, table 5, green cells; electronic supplementary material, table S4). These observations indicate that the change in body proportion that accompanies a change in food concentration depends on the P : C ratio of the diet, and for males at least, vice versa.

Consistent with male and female traits having different nutritional sensitivities, the allometric vectors for the same environmental variable were different in males and females for both food concentration allometries and P : C allometries (Fisher's method, *p* < 0.0025 for both, table 5, red cells; electronic supplementary material, table S5).

As detailed above, because there was a considerable amount of size variation among flies reared in the same vial, the allometric coefficients were calculated from the mean trait sizes for each replicate vial at each P : C ratio and food concentration. However, a few vials generated as few as one individual of either sex, potentially introducing bias to our estimates of mean trait size. This may explain the counterintuitive observation that at very low food concentrations and P : C ratios the male genitalia appeared to show negative relative plasticity in response to changes in P : C ratio and food concentration respectively (figure 1), suggesting that the genitalia increase in size as other traits decrease in size. Consequently, we repeated our analysis only using data from vials that had more than five individuals of the same sex. None of the 1 : 14.2 P : C vials produced sufficient adult flies, and so this P : C ratio was not included in this second analysis. Nevertheless, the results of this second analysis were almost identical to the first (electronic supplementary material, figure S1, tables S6–S10). The only exception was that variation among food-concentration allometries in males was no longer statistically significant (electronic supplementary material, table S9). In this second analysis, the male genitalia still showed a negative allometry when body size varied in response to changes in P : C ratio at a food concentration of 45 g l^−1^ (electronic supplementary material, figure S1).

### 3.3. The effect of diet quality versus quantity on proportion can largely be explained by the individual effects of carbohydrate and protein

The observation that changes in diet quality affect body proportion differently from changes in diet quantity suggests that the developmental mechanisms that regulate the response to each are at least partially distinct. It is possible, however, that rather than responding to food concentration and P : C ratio *per se*, developing larvae are responding to the individual level of protein and carbohydrate in their diet. To test this, we reanalysed the data using a nutritional geometry approach [52], exploring how trait size responded to changes in protein and carbohydrate concentration.

**Figure 3.**
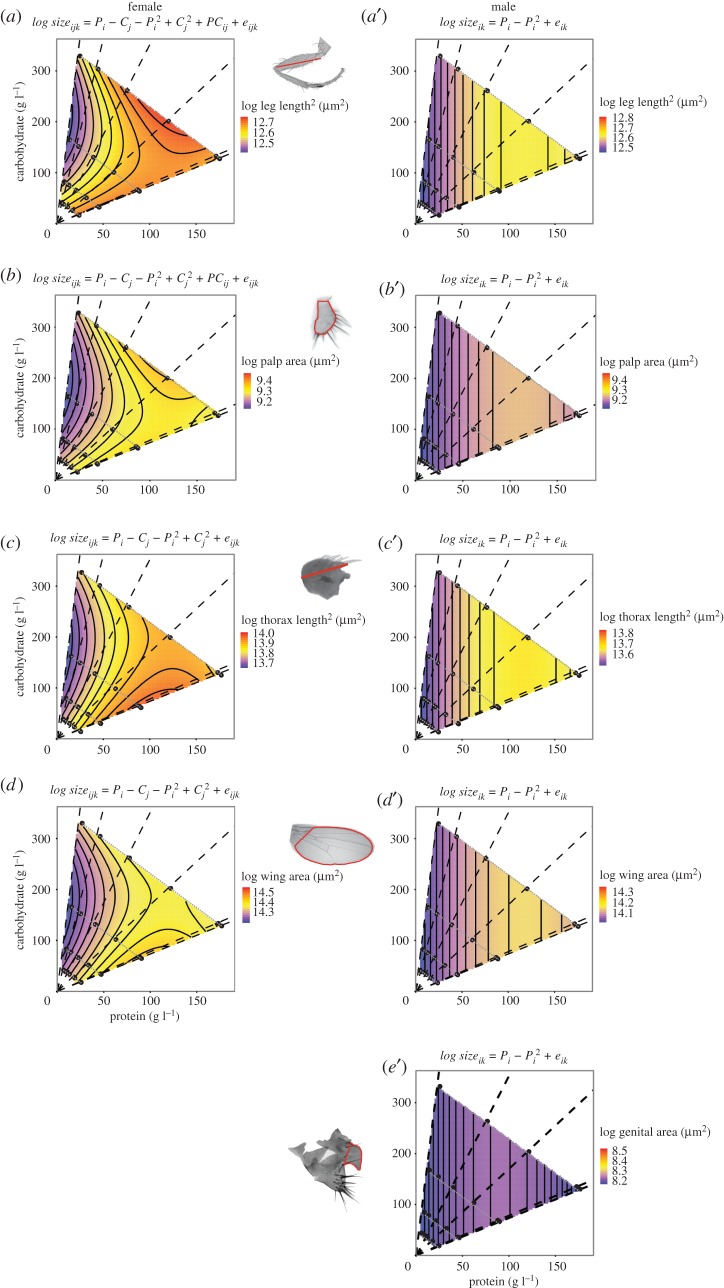
The nutritional geometries of trait size in female and male *Drosophila melanogaster*. Surfaces show the fitted relationship between trait size, carbohydrate level and protein level for (*a*,*a*′) wing, (*b*,*b*′) thorax, (*c*,*c*′) palp and (*d*,*d*′) femur, in females (*a*,*b*,*c*,*d*) and males (*a*′,*b*′,*c*′,*d*′), as well the male genitalia (*e*′), based on the statistical model specified by the equation above each chart (see the electronic supplementary material, table S10, for parameter details). Dashed black lines connect points with equal P : C ratio. Dotted grey lines connect points of equal food concentration. Morphological measurements are shown in the images of each trait.

The specific effect of protein and carbohydrate concentration on size varied with trait and with sex (figure 3). For all traits in both sexes, there was a negative quadratic relationship between trait size and protein level, such that both high and low levels of proteins significantly reduced trait size (figure 3 and table 6). However, while carbohydrate concentration had no detectable effect on male trait size, for all female traits there was also a positive quadratic relationship between carbohydrate level and trait size, such that intermediate levels of carbohydrates reduced trait size (figure 3 and table 6). For the female wing and thorax, protein and carbohydrate level acted additively, while for the female palp and femur there was a small but significant interaction between the effect of protein and carbohydrate on trait size (table 6). Thus, for all male traits, the contrasting effects of diet quality versus diet quantity on body proportion are wholly explained by protein concentration. For female traits, by contrast, carbohydrate concentration also plays a role.

**Table 6.** Effects of carbohydrate (*C*), protein (*P*) and their squares and products on trait size in females. For all traits, the data were fitted with linear mixed-effects models by restricted maximum likelihood. Non-significant parameters were removed from final model, unless their removal rendered the higher order factors non-significant. *p < 0.05, **p < 0.01, ***p < 0.001. Marginal *R^2^* is based on fixed effects only.

body part	sex		*C*	*P*	*C^2^*	*P^2^*	*C × P*	marginal *R^2^*
femur	male	*β*		0.0036		−0.000015		0.224
		*t*-value		4.624***		−3.552***		
	female	*β*	−0.0032	0.0027	0.00001	−0.00002	0.000015	0.388
		*t*-value	−6.669***	3.623***	5.018***	−4.796***	3.450**	
palp	male	*β*		0.0024		−0.00001		0.085
		*t*-value		3.089**		−2.4920**		
	female	*β*	−0.002013	0.002462	0.00001	−0.000015	0.000008	0.220
		*t*-value	−4.392***	3.494**	3.444***	−4.239**	2.13333*	
thorax	male	*β*		0.0038		−0.00002		0.222
		*t*-value		5.031***		−3.874***		
	female	*β*	−0.0020	0.0052	0.00001	−0.00002		0.384
		*t*-value	−4.486***	7.744***	3.609**	−5.594***		
wing	male	*β*		0.0028		−0.000003		0.208
		*t*-value		5.058***		−3.918***		
	female	*β*	−0.0016	0.0038	0.000004	−0.000016		0.372
		*t*-value	−4.845***	7.585***	4.137**	−5.710***		
genital	male	*β*		0.0017		−0.000009		0.030
		*t*-value		3.209**		−3.298**		

## 4. Discussion

The effect of nutrition on body and trait size has been the subject of intense research for well over 100 years, and we have an increasingly comprehensive understanding of the molecular-genetic and physiological mechanisms that regulate the nutritional plasticity of body and trait size [1,30], and how selection acts on these mechanisms to generate a particular body proportion at a particular body size [53]. However, these studies have largely concentrated on nutritional quantity, and we have only a rudimentary understanding of how nutritional quality affects trait size and body proportion. The goal of our study was therefore to test whether traits vary in their size response to changes in diet quality and whether the pattern of this response is the same as the response to changes in diet quantity. Our results suggest that the morphological response to changes in diet quality is different from the response to changes in diet quantity. These divergent effects can be explained by the response of individual traits to the largely additive effects of carbohydrate and protein level.

### 4.1. The morphological response to changes in diet quality and quantity

Previous studies show that an excess of carbohydrates or proteins negatively impacts various life-history characteristics in *Drosophila*. All other dietary components being equal, both high carbohydrate and high protein diets reduce larval size at wandering, adult body size, egg-to-pupal survival and ovariole number in females, but increase total developmental time [23,54,55]. However, the effects of excess carbohydrates or proteins are not always negative. High carbohydrate diets increase lifespan, lifetime egg production and egg production rate, while high protein diets decrease lifespan and lifetime egg production, but increase the rate of egg production [22,56–58]. Thus, the effects of dietary imbalance appear to be more complex than the effects of dietary restriction, which generally has a negative impact on all aspects of life history, except longevity [59]. This complexity is increased by inconsistency among studies in the effects of dietary imbalance. For example, other researchers have found that high protein diets may accelerate development with no effect on body size [9], while excess carbohydrates decrease lifespan [60] and lifetime egg production [57].

Our data indicate that dietary imbalance negatively impacts trait size in *Drosophila*, and are therefore consistent with studies that show the same effect on the size of the body as a whole [23]. Furthermore, for all female traits and the male femur and wing, we observed a negative interaction between diet quality and quantity. That is, the effect of dietary imbalance depends on the level of food concentration. These interactions may explain inconsistencies among studies on the effect of protein-to-carbohydrate ratio on life-history characteristics.

Earlier studies have also demonstrated that, in *Drosophila*, traits vary in their sensitivity to environmental regulators of size, including the level of developmental nutrition [26,31]. Our data indicate that there is also variation among traits in their sensitivity to changes in P : C ratio. In particular, male genital size was only marginally affected by changes in diet quality, consistent with a large number of studies that show male genital size is largely invariant within species (reviewed in [61]). This variation in the relative plasticity among traits means that body proportion changes with size [4]. In general, in both males and females, we found that traits that were more sensitive to changes in diet quality were also more sensitive to changes in diet quantity. Nevertheless, the pattern of relative plasticities among traits was not identical for P : C ratio versus food concentration, and this means that the change in body proportion in response to changes in diet quality was not the same as the change in body proportion in response to changes in diet quantity.

We have previously interpreted the divergent effects of different environmental factors on body proportion as indicating that these environmental factors affect body and trait size through different developmental mechanisms [31]. It would be tempting to conclude that the developmental mechanisms that regulate trait size with respect to diet quality are at least partially distinct from those that regulate trait size with respect to diet quantity. However, by reanalysing our data in the context of protein and carbohydrate concentration, it becomes clear that, for most traits, the independent effects of these two macronutrients are sufficient to explain the effect of diet quality and quantity on trait size. This is most obvious in males, where protein concentration but not carbohydrate concentration affected trait size. Consequently, the differing effects of P : C ratio and food concentration on body proportion in males must be a consequence of variation among traits in their sensitivity to changes in protein concentration alone. In contrast to males, trait size in females was affected by both protein and carbohydrate concentration, but in opposite directions: trait size was maximum at intermediate levels of protein but minimum at intermediate levels of carbohydrates. For the female thorax and wing, carbohydrate and protein level acted additively. This means that the difference in scaling between thorax and wing when P : C ratio changes versus when food concentration changes (figure 1) can also be explained by the individual effects of protein and carbohydrate on trait size. Only for the palp and femur size was there a small but significant interaction between the effects of protein and carbohydrates. Collectively, these data, therefore, suggest that growing flies do not have developmental mechanisms that respond to diet quality directly, but rather respond to the individual levels of macronutrients in their diet.

### 4.2. The effect of carbohydrate and protein level on trait size

Given that there is variation within and between traits in their response to protein and carbohydrate level, and that this variation generates differences in body proportion when body size varies in response to changes in diet quality versus quantity, it is important to understand how different macronutrients affect trait growth, and the mechanisms that underlie this response. Hitherto, most research on the molecular mechanisms that regulate trait size with respect to diet has focused on the mechanisms by which dietary restriction impact morphology and physiology, which is highly conserved among all animals. At a cellular level, the response to developmental nutrition is mediated by the IGF/insulin-like signalling (IIS) pathway and the target of rapamycin (TOR) signalling pathway, collectively referred to as IIS/TOR [1,62,63]. Although there is considerable crosstalk between both the IIS and TOR signalling pathways, they are activated by different mechanisms: IIS is activated by circulating insulin-like peptides (ILPs) released (in part) in response to nutritional levels [64,65] while TOR is activated by cellular levels of amino acids [66] and cellular energy (adenosine triphosphate) [67]. Consequently, the two pathways may have different sensitivities to changes in dietary carbohydrates versus protein, allowing the sensitivity of trait size to each macronutrient to be tuned somewhat independently.

Only a few studies, however, have explored how changes in the macronutrient levels affect signalling through the IIS and TOR pathways. High sugar diets result in reduced insulin sensitivity in peripheral tissue in *Drosophila* larvae [54,68], and this is accompanied by a general reduction in the activity of the insulin-signalling pathway [54,68]. Similarly, in adult flies high sugar diets reduce insulin sensitivity [60], and both low and high carbohydrate levels appear to suppress IIS activity [69]. The effect of protein levels on signalling through the IIS and TOR pathway has been less well elucidated, despite protein having a more potent effect on trait and body size (this study) [23]. A recent study of the nutritional geometry of IIS gene expression in adult *Drosophila* [45] included two genes that are negatively transcriptionally regulated by IIS. They found one, *4EBP*, was only upregulated at very low protein and carbohydrate levels, while the other, *InR*, was primarily upregulated at high protein levels, but appeared unaffected by carbohydrate levels. Further studies exploring how protein levels affect IIS and TOR signalling are clearly necessary if we are to fully understand how macronutrient balance affects phenotype.

### 4.3. Sex-specific nutritional plasticity

Regardless of the patterns of relative sensitivity to changes in diet quantity and quality among traits within males and females, female traits were generally more responsive to changes in diet than male traits. These data are consistent with earlier studies that show female body size is more sensitive to dietary restriction than male body size [24,58,70,71]. Recent studies suggest that the sex-specific difference in the response to nutritional deprivation reflects difference in the nutritional regulation of IIS in females versus males. Nutritionally deprived males and females are the same size [24], as are males and females in which the IIS has been suppressed [71]. In contrast, well-fed females are larger than males and have higher levels of IIS activity, in part because females appear to release dILP2 whereas well-fed males do not [24]. Thus, the higher level of nutritional plasticity in females appears to correspond to the increased nutritional sensitivity of dILP2 release in females relative to males [24].

Our data add an intriguing perspective to this story. The results suggest that, although female traits are marginally more sensitive to changes in protein level than male traits, the primary reason for the elevated nutritional plasticity in females is that they respond to changes in carbohydrate level, while males do not (or at least not detectably in our experiment). This is consistent with an earlier study that detected an effect of carbohydrates on adult body mass in females but not males [23]. If sex-specific differences in nutritional plasticity are due to differences in the plasticity of dILP2 release, then this suggests that dILP2 release is sensitive to dietary carbohydrates in females but not males. There is some evidence that *dILP2* expression does not respond to yeast in adult flies [72,73], and is elevated at low P : C ratios [45], supporting the hypothesis that dILP2 is regulated by carbohydrates rather than protein. However, similar experiments have not yet been conducted in larvae.

## 5. Conclusion

Collectively, our data indicate that the morphological response to changes in diet quality is different from the response to changes in diet quantity and that these responses are sex specific. Consequently, body proportion, and hence body shape, may be different when body size is reduced through a reduction in food concentration versus through a change in P : C ratio. However, our geometric analysis suggests that, rather than responding to food quality and quantity directly, flies are responding to the concentration of protein and carbohydrate in their diet, and that these two macronutrients act largely additively to regulate trait size. That is, it is not the balance of proteins to carbohydrates that is of primary importance, but the concentration of each. Further research into the mechanisms that regulate trait size with respect to the independent effects of carbohydrate and protein is therefore necessary to understand how nutritional variation in body size and proportion is generated and evolves, both between sexes and between species.

## Supplementary Material

Supplementary Figure

## Supplementary Material

Supplementary Tables

## Data accessibility

All data collected for this research, and the R scripts used to analyse them, are deposited in Figshare: https://figshare.com/s/e17afa5d21ffbb1daf69.
