# Supplementary material for: The sex-specific effects of diet quality versus quantity on morphology in Drosophila melanogaster
Source: R Soc Open Sci. 2017 Sep 6;4(9):170375. doi: 10.1098/rsos.170375 (PMC5627086; doi:10.1098/rsos.170375)
Supplement: Supplementary Figure [file rsos170375supp1.pdf]

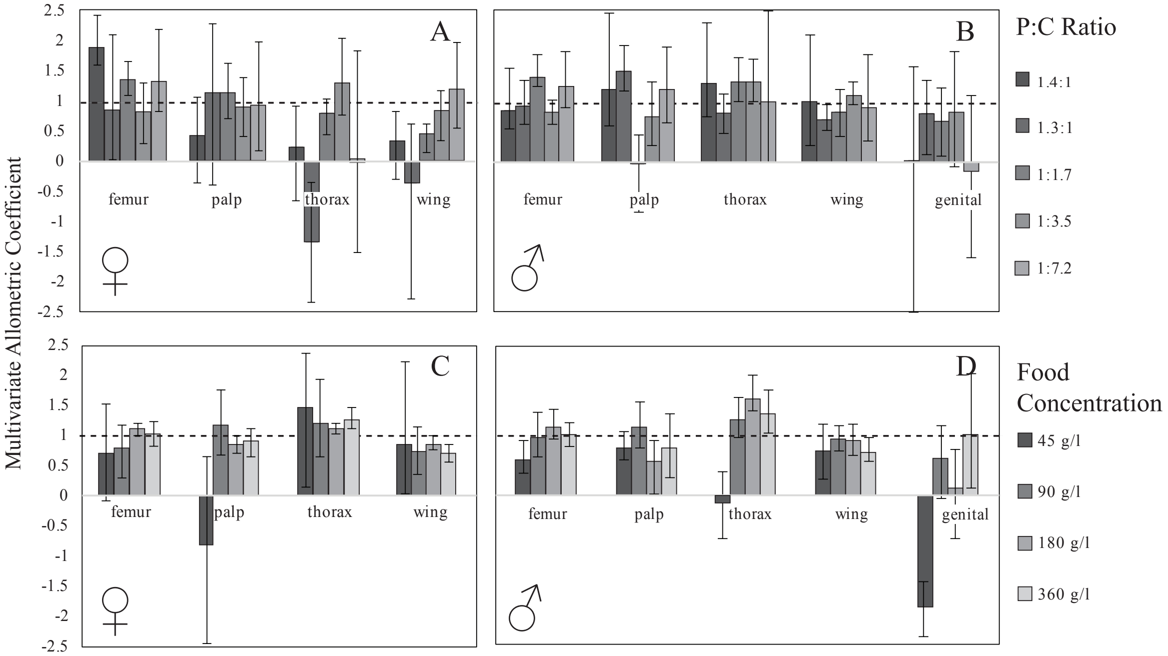


**Supplementary Figure S1:** Multivariate allometric coefficients for female and male traits when size varies with food concentration at different P:C ratios (A, B) and with P:C ratio at different food concentrations (C, D), using only data from vials with more than five individuals of the same sex. The allometric coefficients are standardized such that a coefficient of 1 indicates a trait scales isometrically to body size (horizontal dashed line). Error bars are 95% confidence intervals calculated from 1000 bootstrap samples.
