# Supplementary material for: The sex-specific effects of diet quality versus quantity on morphology in Drosophila melanogaster
Source: R Soc Open Sci. 2017 Sep 6;4(9):170375. doi: 10.1098/rsos.170375 (PMC5627086; doi:10.1098/rsos.170375)
Supplement: Supplementary Tables [file rsos170375supp2.pdf]

Supplementary Table S1: Sample size (N) for each experimental diet.

P:C Ratio	Diet	Sex[1]	Number
(1:14.6)	360 g/l	F	1
(1:14.6)	90 g/l	F	6
(1:7.2)	180 g/l	F	8
(1:7.2)	360 g/l	F	11
(1:7.2)	90 g/l	F	8
(1:3.5)	180 g/l	F	18
(1:3.5)	360 g/l	F	34
(1:3.5)	45 g/l	F	2
(1:3.5)	90 g/l	F	15
(1:1.7)	180 g/l	F	24
(1:1.7)	360 g/l	F	26
(1:1.7)	45 g/l	F	1
(1:1.7)	90 g/l	F	27
(1.3:1)	180 g/l	F	26
(1.3:1)	360 g/l	F	26
(1.3:1)	45 g/l	F	4
(1.3:1)	90 g/l	F	54
(1.4:1)	180 g/l	F	29
(1.4:1)	360 g/l	F	25
(1.4:1)	45 g/l	F	14
(1.4:1)	90 g/l	F	54

P:C Ratio	Diet	Sex[1]	Number
(1:14.6)	180 g/l	M	2
(1:14.6)	90 g/l	M	6
(1:7.2)	180 g/l	M	6
(1:7.2)	360 g/l	M	14
(1:7.2)	90 g/l	M	12
(1:3.5)	180 g/l	M	20
(1:3.5)	360 g/l	M	21
(1:3.5)	90 g/l	M	16
(1:1.7)	180 g/l	M	27
(1:1.7)	360 g/l	M	22
(1:1.7)	90 g/l	M	16
(1.3:1)	180 g/l	M	27
(1.3:1)	360 g/l	M	16
(1.3:1)	45 g/l	M	15
(1.3:1)	90 g/l	M	59
(1.4:1)	180 g/l	M	16
(1.4:1)	360 g/l	M	20
(1.4:1)	45 g/l	M	7
(1.4:1)	90 g/l	M	30

[1] M = male, F = female

Supplementary Table S2: *P*-values for pairwise contrast of allometric vectors between food-concentration allometries and P:C allometries, within each sex.

Females		Males	
Contrast[1]	*P*-value	Contrast	*P*-value
♀1.4:1 v. ♀45 g/l	0.5736	♂1.4:1 v. ♂45 g/l	0.1851
♀1.4:1 v. ♀90 g/l	**0.001**	♂1.4:1 v. ♂90 g/l	0.7416
♀1.4:1 v. ♀180 g/l	**0.0151**	♂1.4:1 v. ♂180 g/l	0.4728
♀1.4:1 v. ♀360 g/l	0.1145	♂1.4:1 v. ♂360 g/l	0.6233
♀1.3:1v. ♀45 g/l	**0.0278**	♂1.3:1v. ♂45 g/l	0.1699
♀1.3:1v. ♀90 g/l	**0.0005**	♂1.3:1v. ♂90 g/l	0.1076
♀1.3:1v. ♀180 g/l	**0.0120**	♂1.3:1v. ♂180 g/l	**0.0152**
♀1.3:1v. ♀360 g/l	**0.0089**	♂1.3:1v. ♂360 g/l	**0.0418**
♀1:1.7 v. ♀45 g/l	0.2879	♂1:1.7 v. ♂45 g/l	0.1273
♀1:1.7 v. ♀90 g/l	**0.0078**	♂1:1.7 v. ♂90 g/l	0.7077
♀1:1.7 v. ♀180 g/l	**0.0180**	♂1:1.7 v. ♂180 g/l	0.1606
♀1:1.7 v. ♀360 g/l	0.0518	♂1:1.7 v. ♂360 g/l	0.4288
♀1:3.5 v. ♀45 g/l	0.5783	♂1:3.5 v. ♂45 g/l	0.1277
♀1:3.5 v. ♀90 g/l	0.1481	♂1:3.5 v. ♂90 g/l	0.1747
♀1:3.5 v. ♀180 g/l	0.0975	♂1:3.5 v. ♂180 g/l	0.0925
♀1:3.5 v. ♀360 g/l	0.2406	♂1:3.5 v. ♂360 g/l	0.4657
♀1:7.2 v. ♀45 g/l	0.0551	♂1:7.2 v. ♂45 g/l	**0.0137**
♀1:7.2 v. ♀90 g/l	**0.0302**	♂1:7.2 v. ♂90 g/l	0.6201
♀1:7.2 v. ♀180 g/l	**0.0377**	♂1:7.2 v. ♂180 g/l	0.0876
♀1:7.2 v. ♀360 g/l	**0.0153**	♂1:7.2 v. ♂360 g/l	0.0950
♀1:14.6 v. ♀45 g/l	0.3980	♂1:14.6 v. ♂45 g/l	0.1441
♀1:14.6 v. ♀90 g/l	0.7136	♂1:14.6 v. ♂90 g/l	0.1106
♀1:14.6 v. ♀180 g/l	0.4007	♂1:14.6 v. ♂180 g/l	**0.0038**
♀1:14.6 v. ♀360 g/l	0.3231	♂1:14.6 v. ♂360 g/l	**0.0118**
**Fisher's P**	**<0.0001**	**Fisher's P**	**<0.0001**

Values in bold and in red cells are significant at $P < 0.05$.

[1] Allometries being compared. For food-concentration allometries the P:C ratio is given, for P:C allometries the food-concentration is given. ♀ is female and ♂ is male. Thus "♀1.4:1 v. ♀45 g/l" is the comparison of the female food-concentration allometry at a P:C of 1.4:1 with a female P:C allometry at a food-concentration level of 45 g/l.

Supplementary Table S3: *P*-values for meta-analysis of pairwise contrast of allometric vectors among P:C allometries, within each sex.

Females		Males	
Contrast[1]	*P*-value	Contrast[1]	*P*-value
♀45 g/l v. ♀90 g/l	0.6972	♂45 g/l v. ♂90 g/l	0.1163
♀45 g/l v. ♀180 g/l	0.7871	♂45 g/l v. ♂180 g/l	0.0868
♀45 g/l v. ♀360 g/l	0.8921	♂45 g/l v. ♂360 g/l	**0.0001**
♀90 g/l v. ♀180 g/l	0.5959	♂90 g/l v. ♂180 g/l	0.2864
♀90 g/l v. ♀360 g/l	0.5230	♂90 g/l v. ♂360 g/l	0.2226
♀180 g/l v. ♀360 g/l	0.3529	♂180 g/l v. ♂360 g/l	0.2004
**Fisher's P**	0.9234	**Fisher's P**	**0.0003**

Values in bold and in red cells are significant at $P < 0.05$.

[1] Allometries being compared. For P:C allometries the food- concentration is given. ♀ is female and ♂ is male. Thus "♀45 g/l v. ♀90 g/l" is the comparison of female P:C allometries at a food-concentration level of 45 g/l versus 90 g/l.

Supplementary Table S4: *P*-values for meta-analysis of pairwise contrast of allometric vectors among food-concentration allometries, within each sex.

Females		Males	
Contrast[1]	*P*-value	Contrast[1]	*P*-value
♀1.4:1 v. ♀1:1	0.1499	♂1.4:1 v. ♂1:1	0.5462
♀1.4:1 v. ♀1:1.7	**0.0343**	♂1.4:1 v. ♂1:1.7	0.6206
♀1.4:1 v. ♀1:3.5	**0.0082**	♂1.4:1 v. ♂1:3.5	0.4929
♀1.4:1 v. ♀1:7.2	**0.0004**	♂1.4:1 v. ♂1:7.2	0.7699
♀1.4:1 v. ♀1:14.6	0.1570	♂1.4:1 v. ♂1:14.6	0.1007
♀1.3:1 v. ♀1:1.7	**0.0071**	♂1.3:1 v. ♂1:1.7	0.1521
♀1.3:1 v. ♀1:3.5	**0.0001**	♂1.3:1 v. ♂1:3.5	0.0713
♀1.3:1 v. ♀1:7.2	**0.0012**	♂1.3:1 v. ♂1:7.2	0.1058
♀1.3:1 v. ♀1:14.6	0.0668	♂1.3:1 v. ♂1:14.6	**0.0076**
♀1:1.7 v. ♀1:3.5	**0.0115**	♂1:1.7 v. ♂1:3.5	0.5473
♀1:1.7 v. ♀1:7.2	0.0615	♂1:1.7 v. ♂1:7.2	0.2072
♀1:1.7 v. ♀1:14.6	0.1378	♂1:1.7 v. ♂1:14.6	0.0645
♀1:3.5 v. ♀1:7.2	**0.0049**	♂1:3.5 v. ♂1:7.2	0.1459
♀1:3.5 v. ♀1:14.6	0.2789	♂1:3.5 v. ♂1:14.6	0.1836
♀1:7.2 v. ♀1:14.6	0.4992	♂1:7.2 v. ♂1:14.6	0.0663
**Fisher's P**	<**0.0001**	**Fisher's P**	**0.0040**

Values in bold and in red cells are significant at $P < 0.05$.

[1] Allometries being compared. For food-concentration allometries the P:C ratio is given. ♀ is female and ♂ is male. Thus "♀1.4:1 v. ♀1:1" is the comparison of female food-concentration allometries at a P:C level of 1.4:1 versus 1:1.

Supplementary Table S5: *P*-values for pairwise contrast of allometric vectors between males and female P:C allometries and food-concentration allometries.

Food-concentration Allometries	
Contrast[1]	*P*-value
♀ 1.4:1 v. ♂ 1.4:1	**0.0001**
♀ 1.3:1 v. ♂ 1.3:1	**0.0002**
♀ 1:1.7 v. ♂ 1:1.7	0.7562
♀ 1:3.5 v. ♂ 1:3.5	0.1286
♀ 1:7.2 v. ♂ 1:7.2	0.1683
♀ 1:14.6 v. ♂ 1:14.6	0.1511
**Fisher's P**	<**0.0001**

Food-Concentration Allometries	
Contrast[1]	*P*-value
♀ 45 g/l v. ♂ 45 g/l	**0.0352**
♀ 90 g/l v. ♂ 90 g/l	0.8323
♀ 180 g/l v. ♂ 180 g/l	0.1401
♀ 360 g/l v. ♂ 360 g/l	**0.0017**
**Fisher's P**	**0.0025**

Values in bold and in red cells are significant at $P < 0.05$.

[1] Allometries being compared. For food-concentration allometries the P:C ratio is given, for P:C allometries the food concentration is given. ♀ is female and ♂ is male. Thus "♀ 1.4:1 v. ♂ 1.4:1" is the comparison of the female and male food-concentration allometries at a P:C of 1.4:1.

Supplementary Table S6: Pairwise comparisons of allometries generated in response to variation in caloric value (food-concentration allometries) at different P:C ratios and in response to variation in P:C ratio (P:C allometries) at different food concentrations, in males (♂) and females (♀), using only data from vials with >5 flies of the same sex.

		Food-concentration Allometries										P:C Allometries							
		♂1.4:1	♂1.3:1	♂1:1.7	♂1:3.5	♂1:7.2	♀1.4:1	♀1.3:1	♀1:1.7	♀1:3.5	♀1:7.2	♂45 g/l	♂90 g/l	♂180 g/l	♂360 g/l	♀45 g/l	♀90 g/l	♀180 g/l	♀360 g/l
Food-concentration Allometries	♂1.4:1		27.761	39.710	24.328	13.264	50.782	135.248	24.493	7.675	38.048	115.513	16.470	20.092	29.827	56.953	5.865	11.771	10.990
	♂1.3:1	0.409		44.981	26.527	28.441	42.899	140.162	14.853	24.465	29.959	114.264	17.787	38.698	25.042	72.782	16.875	21.115	21.897
	♂1:1.7	0.139	0.218		26.553	40.122	38.631	101.401	37.045	29.477	46.532	111.235	32.055	23.220	24.813	30.450	37.509	26.059	27.531
	♂1:3.5	0.419	0.344	0.363		31.380	50.607	127.020	30.201	7.951	39.909	127.622	12.103	22.516	12.031	46.034	15.902	11.665	12.684
	♂1:7.2	0.727	0.556	0.164	0.301		38.955	132.337	13.630	15.378	28.482	102.777	22.236	24.044	34.550	60.626	12.624	9.113	12.121
	♀1.4:1	**0.023**	**0.000**	**0.029**	**0.054**	**0.456**		102.015	30.727	49.315	33.785	80.346	47.314	46.312	44.837	65.974	49.933	40.318	43.424
	♀1.3:1	**0.005**	**0.090**	**0.000**	**0.000**	**0.000**	0.000		125.387	127.723	130.485	72.780	132.555	115.801	120.723	84.496	134.499	126.140	125.201
	♀1:1.7	**0.073**	0.040	**0.007**	**0.047**	**0.665**	**0.118**	**0.000**		25.291	31.378	99.249	21.355	30.108	23.766	66.519	21.541	18.889	19.464
	♀1:3.5	**0.345**	**0.136**	0.019	0.158	**0.446**	**0.110**	**0.000**	**0.092**		41.519	124.492	6.481	12.597	7.608	50.418	9.475	9.571	6.612
	♀1:7.2	0.680	0.767	0.557	0.385	0.542	**0.480**	**0.000**	**0.362**	0.109		93.258	36.816	45.165	42.933	71.586	40.068	33.469	39.205
P:C Allometries	♂45 g/l	0.000	0.000	0.000	0.000	**0.000**	0.099	0.087	0.000	**0.000**	0.000		120.777	114.787	124.043	133.410	119.265	116.587	118.473
	♂90 g/l	0.176	0.098	0.013	0.471	0.500	**0.000**	**<0.001**	**0.002**	**0.452**	0.414	0.000		22.098	14.737	56.052	5.618	8.457	7.629
	♂180 g/l	0.336	**0.258**	0.051	0.307	0.431	**0.012**	**<0.001**	**0.001**	0.100	0.541	0.000	0.780		25.623	39.026	21.541	14.080	12.958
	♂360 g/l	0.353	**0.155**	0.259	0.408	0.472	**0.001**	**<0.001**	**<0.001**	0.411	0.675	**0.000**	0.194	0.247		46.801	14.652	9.605	5.595
	♀45 g/l	**0.172**	**0.178**	0.217	**0.092**	0.000	0.152	**0.074**	0.073	0.220	0.000	**0.000**	0.085	0.145	0.263		59.855	51.679	51.973
	♀90 g/l	**0.303**	**0.006**	0.038	0.149	0.732	**0.009**	**0.000**	**0.202**	0.805	**0.380**	0.000	0.231	0.030	0.007	0.347		13.258	10.278
	♀180 g/l	0.087	**0.009**	0.010	**0.088**	0.784	**0.040**	**0.000**	**0.036**	0.534	**0.271**	**0.000**	0.079	0.016	**0.058**	0.233	0.271		6.422
	♀360 g/l	0.051	**0.000**	0.011	**0.063**	0.685	0.167	**0.000**	0.279	0.652	**0.268**	0.000	0.084	0.005	**0.286**	0.341	0.570	0.629	

Angles between allometric vectors are shown above the diagonal and uncorrected *P*-values are shown below the diagonal. *P*-values <0.05 are shown in bold. The darker the cell the lower the *P*-value. Green cells show comparison among food-concentration allometries within each sex, purple cells show comparison among P:C allometries within each sex, blue cells show comparison between food-concentration and P:C allometries within each sex, and red cells shows comparisons between male and female allometries.

Supplementary Table S7: *P*-values for pairwise contrast of allometric vectors between food-concentration allometries and P:C allometries within each sex, using only data from vials with >5 flies of the same sex.

Females	
Contrast[1]	*P*-value
♀1.4:1 v. ♀45 g/l	0.152
♀1.4:1 v. ♀90 g/l	**0.009**
♀1.4:1 v. ♀180 g/l	**0.040**
♀1.4:1 v. ♀360 g/l	0.167
♀1.3:1 v. ♀45 g/l	0.074
♀1.3:1 v. ♀90 g/l	**0.001**
♀1.3:1 v. ♀180 g/l	**0.001**
♀1.3:1 v. ♀360 g/l	**0.001**
♀1:1.7 v. ♀45 g/l	0.073
♀1:1.7 v. ♀90 g/l	0.202
♀1:1.7 v. ♀180 g/l	**0.036**
♀1:1.7 v. ♀360 g/l	0.279
♀1:3.5 v. ♀45 g/l	0.220
♀1:3.5 v. ♀90 g/l	0.805
♀1:3.5 v. ♀180 g/l	0.534
♀1:3.5 v. ♀360 g/l	0.652
♀1:7.2 v. ♀45 g/l	**0.001**
♀1:7.2 v. ♀90 g/l	0.380
♀1:7.2 v. ♀180 g/l	0.271
♀1:7.2 v. ♀360 g/l	0.268
Fisher's P	**<0.0001**

Males	
Contrast[1]	*P*-value
♂1.4:1v. ♂45 g/l	**0.001**
♂1.4:1v. ♂90 g/l	0.176
♂1.4:1v. ♂180 g/l	0.336
♂1.4:1v. ♂360 g/l	0.353
♂1.3:1v. ♂45 g/l	**0.001**
♂1.3:1v. ♂90 g/l	0.098
♂1.3:1v. ♂180 g/l	0.258
♂1.3:1v. ♂360 g/l	0.155
♂1:1.7 v. ♂45 g/l	**0.001**
♂1:1.7 v. ♂90 g/l	**0.013**
♂1:1.7 v. ♂180 g/l	0.051
♂1:1.7 v. ♂360 g/l	0.259
♂1:3.5 v. ♂45 g/l	**0.001**
♂1:3.5 v. ♂90 g/l	0.471
♂1:3.5 v. ♂180 g/l	0.307
♂1:3.5 v. ♂360 g/l	0.408
♂1:7.2 v. ♂45 g/l	**0.001**
♂1:7.2 v. ♂90 g/l	0.500
♂1:7.2 v. ♂180 g/l	0.431
♂1:7.2 v. ♂360 g/l	0.472
Fisher's P	**<0.0001**

Values in bold and in red cells are significant at *P*< 0.05.

[1] Allometries being compared. For food-concentration allometries the P:C ratio is given, for P:C allometries the food concentration is given. ♀ is female and ♂ is male. Thus "♀1.4:1 v. ♀45 g/l" is the comparison of the female food-concentration allometry at a P:C of 1.4:1 with a female P:C allometry at a food-concentration level of 45 g/l.

Supplementary Table S8: *P*-values for meta-analysis of pairwise contrast of allometric vectors among P:C allometries within each sex, using only data from vials with >5 flies of the same sex.

Female	
Contrast[1]	*P*-value
♀45g v. ♀90g	0.347
♀45g v. ♀180g	0.233
♀45g v. ♀360g	0.341
♀90g v. ♀180g	0.271
♀90g v. ♀360g	0.570
♀180g v. ♀360g	0.629
Fisher's P	0.4582

Male	
Contrast[1]	*P*-value
♂45g v. ♂90g	**<0.001**
♂45g v. ♂180g	**<0.001**
♂45g v. ♂360g	**<0.001**
♂90g v. ♂180g	0.780
♂90g v. ♂360g	0.194
♂180g v. ♂360g	0.247
Fisher's P	**<0.0001**

Values in bold and in red cells are significant at $P < 0.05$.

[1] Allometries being compared. For P:C allometries the food- concentration is given. ♀ is female and ♂ is male. Thus "♀45 g/l v. ♀90 g/l" is the comparison of female P:C allometries at a food-concentration level of 45 g/l versus 90 g/l.

Supplementary Table S9: *P*-values for meta-analysis of pairwise contrast of allometric vectors among food-concentration allometries within each sex, using only data from vials with >5 flies of the same sex.

Females		Males	
Contrast[1]	*P*-value	Contrast[1]	*P*-value
♀ 1.4:1 v. ♀ 1.3:1	**<0.001**	♂1.4:1 v. ♂1.3:1	0.409
♀ 1.4:1 v. ♀ 1:1.7	0.118	♂1.4:1 v. ♂1:1.7	0.139
♀ 1.4:1 v. ♀ 1:3.5	0.110	♂1.4:1 v. ♂1:3.5	0.419
♀ 1.4:1 v. ♀ 1:7.2	0.480	♂1.4:1 v. ♂1:7.2	0.727
♀ 1.3:1 v. ♀ 1:1.7	**<0.001**	♂1.3:1 v. ♂1:1.7	0.218
♀ 1.3:1 v. ♀ 1:3.5	**<0.001**	♂1.3:1 v. ♂1:3.5	0.344
♀ 1.3:1 v. ♀ 1:7.2	**<0.001**	♂1.3:1 v. ♂1:7.2	0.556
♀ 1:1.7 v. ♀ 1:3.5	0.092	♂1:1.7 v.♂1:3.5	0.363
♀ 1:1.7 v. ♀ 1:7.2	0.362	♂1:1.7 v.♂1:7.2	0.164
♀ 1:3.5 v. ♀ 1:7.2	0.109	♂1:3.5 v.♂1:7.2	0.301
Fisher's P	<**0.0001**	Fisher's P	0.315

Values in bold and in red cells are significant at $P < 0.05$.

[1] Allometries being compared. For food-concentration allometries the P:C ratio is given. ♀ is female and ♂ is male. Thus "♀ 1.4:1 v. ♀ 1:1" is the comparison of female food-concentration allometries at a P:C level of 1.4:1 versus 1:1.

Supplementary Table S10: *P*-values for pairwise contrast of allometric vectors between males and female P:C allometries and food-concentration allometries within each sex, using only data from vials with >5 flies of the same sex.

Caloric Allometries	
Contrast[1]	P-value
♀ 1.4:1 v. ♂ 1.4:1	**0.023**
♀ 1.3:1 v. ♂ 1.3:1	**0.090**
♀ 1:1.7 v. ♂ 1:1.7	**0.007**
♀ 1:3.5 v. ♂ 1:3.5	0.158
♀ 1:7.2 v. ♂ 1:7.2	0.542
Fisher's P	**0.0024**

P:C Allometries	
Contrast[1]	P-value
♀ 45 g/l v. ♂ 45 g/l	**0.001**
♀ 90 g/l v. ♂ 90 g/l	0.231
♀ 180 g/l v. ♂ 180 g/l	**0.016**
♀ 360 g/l v. ♂ 360 g/l	0.286
Fisher's P	**0.0006**

Values in bold and in red cells are significant at $P < 0.05$.

[1] Allometries being compared. For food-concentration allometries the P:C ratio is given, for P:C allometries the food concentration is given. ♀ is female and ♂ is male. Thus "♀ 1.4:1 v. ♂ 1.4:1" is the comparison of the female and male food-concentration allometries at a P:C of 1.4:1.
